# Targeting ARNT attenuates chemoresistance through destabilizing p38α-MAPK signaling in glioblastoma

**DOI:** 10.1038/s41419-024-06735-1

**Published:** 2024-05-28

**Authors:** Wahafu Alafate, Gen Lv, Jiantao Zheng, Haiping Cai, Wei Wu, Yong Yang, Shichao Du, Dong Zhou, Peng Wang

**Affiliations:** 1 Department of Neurosurgery, Guangdong Provincial People’s Hospital (Guangdong Academy of Medical Sciences), Southern Medical University, Guangzhou, China; 2 Department of Neurosurgery, The First Affiliated Hospital of Xi’an Jiaotong University, Xi’an, Shaanxi China; 3 Center of Brain Science, The First Affiliated Hospital of Xi’an Jiaotong University, Xi’an, Shaanxi China; 4 Department of Neurosurgery, Heyuan People’s Hospital, Heyuan, China

**Keywords:** Cancer therapeutic resistance, Stress signalling

## Abstract

Glioblastoma (GBM) is the most aggressive and lethal brain tumor in adults. This study aimed to investigate the functional significance of aryl hydrocarbon receptor nuclear translocator (ARNT) in the pathogenesis of GBM. Analysis of public datasets revealed ARNT is upregulated in GBM tissues compared to lower grade gliomas or normal brain tissues. Higher ARNT expression correlated with the mesenchymal subtype and poorer survival in GBM patients. Silencing ARNT using lentiviral shRNAs attenuated the proliferative, invasive, and stem-like capabilities of GBM cell lines, while ARNT overexpression enhanced these malignant phenotypes. Single-cell RNA sequencing uncovered that ARNT is highly expressed in a stem-like subpopulation and is involved in regulating glycolysis, hypoxia response, and stress pathways. Mechanistic studies found ARNT activates p38 mitogen-activated protein kinase (MAPK) signaling to promote chemoresistance in GBM cells. Disrupting the ARNT/p38α protein interaction via the ARNT PAS-A domain restored temozolomide sensitivity. Overall, this study demonstrates ARNT functions as an oncogenic driver in GBM pathogenesis and represents a promising therapeutic target.

## Introduction

Glioblastoma (GBM) is the most common and aggressive primary malignant brain tumor with median survival of only 15 months [1, 2]. The current standard treatment for GBM patients consists of maximal safe surgical resection followed by radiotherapy and temozolomide (TMZ) chemotherapy. However, this regimen has only marginally improved median survival from 12 to 16 months [3]. Accumulating evidence shows that acquired chemoresistance to TMZ is the primary cause of treatment failure, leading to eventual tumor recurrence and poor prognosis [4]. The dismal prognosis also stems from limited understanding of GBM pathogenesis and a lack of timely diagnostic and therapeutic monitoring tools. Elucidating the molecular mechanisms underlying GBM development and progression, as well as exploring reliable biomarkers, is therefore critical [5].

Hypoxia has long been implicated in genetic instability and tumor progression. Hypoxia inducible factors (HIFs) are central players in cellular hypoxia adaptation to low oxygen, and the HIF-1 signaling pathway is critical to tumor development and progression [6, 7]. The aryl hydrocarbon receptor nuclear translocator (ARNT), also known as hypoxia-inducible factor (HIF)-1β [8], is a member of the basic-Helix-Loop-Helix PER/ARNT/SIM (bHLH/PAS) protein family. ARNT plays a pivotal role in adaptive responses to environmental stresses like dioxin exposure and oxygen deprivation (hypoxia) [9]. Previous studies have demonstrated that ARNT expression is significantly upregulated in a variety of malignant tumors, including renal cell carcinoma [10], lymphoid cancer [11], multiple myeloma [12], breast cancer [13], and medulloblastoma [14]. Moreover, ARNT has been linked to various aspects of malignant transformation, including tumorigenesis, tumor progression, and therapeutic resistance [15]. Despite these findings implicating ARNT in tumor development and aggression, the specific molecular mechanisms underlying the ARNT-GBM relationship remain to be elucidated.

p38 is a MAP kinase family molecule requiring the phosphorylation of threonine and tyrosine residues in the TGY sequence for its activation [16]; currently, four isoforms containing this sequence including p38α, p38β, p38γ, and p38δ have been identified [17] Most biological functions typically attributed to p38 mitogen-activated protein kinases (MAPKs) specifically involve the p38α isoform, which is encoded by the MAPK14 gene. More than 100 proteins can be directly phosphorylated by p38α, and a large fraction of them are involved in the regulation of gene expression [18, 19]. In addition, the p38α pathway can control the production of extracellular signaling molecules such as cytokines, chemokines, and growth factors at different levels [20]. Recently, p38α is recognized as a potential therapeutic target in tumors [18, 21].

p38 MAPK signaling is a stress sensor that responds to diverse stimuli, including DNA damage, growth factors, starvation, etc. [22]. Emerging evidence suggests a dual role of p38 MAPK in cancer development under distinct contexts and circumstances [23]. The enhanced p38 MAPK phosphorylation level was also found to be associated with malignancy in distinct types of cancers such as non-small cell lung cancer [24], breast cancer [25], and head and neck squamous cell carcinomas [26]. It is reported that p38 modulates the activity of the transcription factor activator protein-1(AP-1), which is one of the most well-described nuclear targets of the MAPK cascades and important for cell proliferation to contribute to malignant transformation [27]. Another study demonstrated that the protein complex of MK2 and p38 phosphorylates cytoplasmic heat shock protein 27 (HSP27), which plays a role in regulating cell migration and invasion in thyroid cancer cells [28]. Approximately 88% of gliomas show changes in the mitogen-activated protein kinase (MAPK) pathway, with MAPK genes determining the choice of invasive or proliferative phenotypes and, therefore, regulating metastases development and type [29, 30]. An important fraction of glioblastoma exhibits high MAPK phosphorylation. Unusual p-MAPK expression is a potent independent prognostic marker for poor overall survival (OS), and results in the increased tumor therapy resistance to radiation therapy or temozolomide [31, 32]. Therefore, it is essential to explore the potential molecular mechanisms of the MAPK pathway in GBM.

In this study, we found that ARNT expression is significantly upregulated in glioblastoma multiforme (GBM) and its higher expression correlates with poorer prognosis. Moreover, ARNT levels were closely associated with increased GBM aggressiveness and temozolomide (TMZ) tolerance. We further demonstrated that ARNT activates the mitogen-activated protein kinase (MAPK) signaling pathway by stabilizing p38α, a key MAPK isoform encoded by MAPK14. Taken together, our results suggest the ARNT-p38α-MAPK axis could represent a promising therapeutic target for GBM treatment.

## Materials and methods

### Differential gene expression analysis

Gene expression datasets and relevant clinical information were extracted from public cancers/glioma datasets, including CGGA [33], Gravendeel [34], Rembrandt [35], and TCGA datasets [36]. The limma package was then utilized for exploring the differentially expressed genes in these datasets [37]. The expression difference of individual gene was defined by log2 (Fold change) and adjusted *p* value, in which the absolute value of log2FC larger than 2 times of average±standard deviation with an adjusted *p*-value < 0.05 was defined as a differentially expressed gene.

### Ethical statement and human tissue samples

The use of human tissue samples and animal experiments in this study was approved by the Scientific Ethics Committee of Guangdong Provincial People’s Hospital (approval no. KY-Z-2022-231-01). Informed consent for tissue donation was obtained from all participating patients. 36 Glioma and non-tumor brain tissue samples were collected in recent years from patients undergoing neurosurgical resection procedures at Guangdong Provincial People’s Hospital.

### qRT-PCR and RNA-seq

Total RNA was extracted using RNeasy mini kits following the manufacturer’s protocol. RNA concentration was quantified by Nanodrop 2000. cDNA was synthesized from the extracted RNA according to standard procedures. qRT-PCR was then performed using ACTB as an internal control gene. Relative mRNA expression levels were calculated using the 2-ΔΔCt method [38]. The primer sequences used were as follows:

ARNT(F):5′-CTGCCAACCCCGAAATGACAT-3′ and ARNT(R):5′- CGCCGCTTAATAGCCCTCTG-3′ [39].

ACTB(F):5′- CATGTACGTTGCTATCCAGGC-3′ and

ACTB(R):5′-CTCCTTAATGTCACGCACGAT-3′ [40].

RNA-seq methodologies and initial analyses were conducted by LC Sciences using Illumina 2000 and 2 × 100 bp paired-end sequencing as previously described [38]. The sequence results were obtained as FPKM (fragment per kilobase of exons per million reads) for each transcript.

### Western blot analysis

Western blot analysis was performed as previously described [4]. Antibodies used in this study were shown as below: Anti-β-actin primary antibody was purchased from Abcam and served as an internal control((cat. No.ab115777). In brief, protein extracts were prepared by lysing cells or tissues in RIPA buffer containing protease and phosphatase inhibitor cocktails (MedChem Express). Protein concentrations were quantified with a BCA assay kit (Thermo Scientific). Equal amounts of protein were denatured by boiling, separated by SDS-polyacrylamide gel electrophoresis, and transferred to polyvinylidene fluoride (PVDF) membranes. The membranes were blocked with 5% skimmed milk and then incubated with primary antibodies followed by HRP-conjugated secondary antibodies. Chemiluminescent signals were detected using ClarityTM Western ECL Substrate (Bio-Rad).Anti-ARNT primary antibody was purchased from Abcam(cat. No. ab270520). Anti-p38α primary antibody was purchased from Abcam (cat. No. ab170099). Anti-p-ERK primary antibody was purchased from Cell signaling technology(cat. No. #8544). Anti-ERK primary antibody was purchased from Cell signaling technology (cat. No. #4695).

### Immunohistochemistry

Immunohistochemistry (IHC) was performed as previously described [4]. Anti-ARNT primary antibody was purchased from Abcam (cat. No. ab270520). Anti-MAPK14 primary antibody was purchased from Abcam (cat. No. ab31828). Goat anti-rabbit IgG purchased from Abcam (cat. No. ab97051) was used as the secondary antibody. Briefly, paraffin-embedded tissue samples were sectioned into 4-μm slices. The sections were deparaffinized, rehydrated, and incubated overnight at 4 °C with primary antibodies for staining. After washing, the slides were incubated with corresponding secondary antibodies and developed using DAB substrate. Finally, hematoxylin counterstaining was performed and images were acquired under a light microscope.

### Cell culture

GBM-derived primary culture cells were obtained as previously described [38]. Fresh surgical GBM specimens were collected and rinsed in PBS to remove blood and necrotic tissue. Mechanical and enzymatic dissociation using trypsin was performed to obtain single-cell suspensions. The isolated single cells were cultured in DMEM-F12 containing 15% fetal bovine serum (FBS), 2% B27 supplement, and 1% penicillin/streptomycin antibiotics at 37 °C with 5% CO2. To validate the quality of the primary cells, they were injected into the brains of nude mice. Primary cells capable of forming tumors in vivo were used for subsequent experiments.

U251MG,SF295,U118MG,A172,T98G,U87MG,Normal Human Astrocytes (NHA) cell lines were purchased from Cell bank, Type culture collection, Chinese Academy of Sciences (Shanghai, China). The cells were cultured in DMEM-F12 medium supplemented with 10% fetal bovine serum and 1% penicillin/streptomycin antibiotics. They were incubated at 37 °C with 5% CO2 in a humidified incubator. To mimic hypoxic conditions, 100 µM CoCl_2_ was used as previously described [41, 42].

### Lentivirus production and transduction

Lentivirus production and transduction were performed as described previously [4]. Lentiviral constructs for ARNT overexpression and knockdown were designed and synthesized by Genechem (Shanghai, China). The GBM cell lines 7209 and U87MG were transduced with ARNT shRNA or scramble shRNA control lentiviruses, while 1763 and U251MG cells were transduced with ARNT overexpression lentivirus or empty vector control, following the manufacturer’s protocol. After transduction, the cells were selected with 0.5 mg/mL puromycin for 4 weeks to obtain stable expression. The shRNA lentiviral constructs targeted the following sequences:

shARNT#1(5′-GGCTCAAGGAGATCGTTTATT-3′),

shARNT#2(5′-ACTAGGTCCCACAGCTAATTT-3′), and the scramble control(5′- TCTCGGCATGGACGAGCTGTA-3′).

### Matrigel invasion assays

The invasion ability of the 1763, 7209, U87MG, and U251MG cell lines was assessed using Matrigel invasion assays. 5 × 10^5^ cells suspended in serum-free DMEM/F12 were seeded into the upper chamber of a transwell insert with 8μm pores (BD Biosciences). The lower chamber contained DMEM/F12 with 10% fetal bovine serum (FBS) as a chemoattractant. After 24 h incubation, cells that had invaded through the Matrigel to the underside of the transwell membrane were fixed with 20% methanol and stained with 0.1% crystal violet. Non-invaded cells were removed from the upper surface of the filters using cotton swabs. Stained cells on the underside of the membrane were imaged under an inverted microscope.

### Sphere formation assay

Sphere formation assays were performed as previously described [38]. Cells were seeded into 24-well ultra-low attachment plates in serum-free DMEM/F12 medium supplemented with 5 μg/ml B27 supplement (1×), 10 ng/ml basic fibroblast growth factor, and 20 ng/ml human recombinant EGF. After 6 days of incubation, the number and size of spheres formed were visualized under an inverted microscope.

### Colony formation assay

Colony formation assays were performed to assess the effect of ARNT on GBM cell proliferation. 1763, 7209, U87MG, and U251MG cells under various treatment conditions were seeded into 6-well plates. After 14 days of culture to allow colony formation, the cells were fixed with methanol and stained with methylene blue.

### Flow cytometry

Flow cytometry assays were conducted as previously described [43]. Cell apoptosis was measured by the Annexin V-PE/7-AAD Cell Apoptosis Detection Kit (Servicebio) strictly following the manufacturer’s protocols.

### In vivo intracranial xenograft tumor model

All animal experiments were approved by the Ethics Committee of Guangdong Provincial People’s Hospital. 6-week-old female nude mice were randomly chosen for the intracranial xenograft model. 1 ×10^5 7209 cells transduced with or without lentivirus in 2 μL PBS were slowly injected into the mouse brains as previously described. 5 mice were used per group. Mice were sacrificed and perfused with ice-cold PBS and 4% paraformaldehyde when they displayed symptoms of unsteady gait, arched back, >10% weight loss, or leg paralysis. The brains were dissected, fixed in 4% paraformaldehyde for 24 h, transferred to 10% formalin, and sectioned.

### Gene set enrichment analysis and Kyoto encyclopedia of genes and genomes analysis

Gene expression profiles were obtained from mRNA sequencing data or public databases as described previously. The data was pre-processed in R software including normalization and gene ID transformation. The limma package was used to identify differentially expressed genes (DEGs) between ARNT knockdown versus control groups. GO annotation and Kyoto encyclopedia of genes and genomes pathway enrichment analysis were performed on the DEGs and results were visualized as a chord diagram and histogram respectively. Additionally, gene set enrichment analysis (GSEA) was carried out according to the software guidelines to identify pathways correlated with ARNT.

### Co-immunoprecipitation

Co-immunoprecipitation (co-IP) assays were performed as previously described [38] using the Pierce Co-IP kit (Thermo Fisher Scientific). Briefly, total cellular proteins were extracted using IP lysis buffer. ARNT antibody (1:50 dilution) or p38α antibody (1:50 dilution) were added to the lysates along with a non-specific IgG antibody control. The antibody-protein complexes were allowed to form overnight at 4 °C. Protein A Dynabeads (Thermo Fisher Scientific) were then added and incubated for 2 h to capture the antibody-protein complexes. The bead-bound immunocomplexes were washed three times with PBST buffer. The immunoprecipitated proteins were then analyzed by western blot as described previously.

### Glutathione S-transferase pull-down assay

Glutathione S-transferase (GST)-tagged ARNT was transfected into HEK293T cells along with or without p38α plasmid using lipofectamine according to the manufacturer’s instructions. After 24 h incubation at 4 °C, cell lysates were collected and incubated with glutathione sepharose beads for 1 h to capture GST-tagged proteins. The GST-bound complexes were washed sufficiently with lysis buffer and analyzed by western blot. The ARNT protein was truncated into four segments based on its predicted 2D structure. Subsequently, plasmids containing the target fragments were inserted into Top10 E.coli cells (Thermo Fisher, Invitrogen) using the Gateway system, following the manufacturer’s instructions. The plasmids were then harvested and purified for use in GST-pulldown assays with 293T cells.

### Single-cell gene expression quantification and subcluster detection

scRNAseq data and relevant clinical data were downloaded from CGGA database [33]. The raw gene expression matrices were imported into R and analyzed using the Seurat package (v2.3.4). Low-quality cells were filtered out based on metrics including the number of unique molecular identifiers (UMIs), the number of expressed genes, and the percentage of mitochondrial UMIs. Gene expression data for the high-quality cells were normalized by total UMI counts and scaled using a regression model to account for confounding sources of variation. Highly variable genes were identified using the Seurat FindVariableGenes function with customized parameters. Principal component analysis (PCA) was performed on the variable genes and significant principal components were selected for dimensionality reduction. Cell clustering was done using the FindClusters function on the reduced PCA dimensions. Finally, t-SNE analysis was used to visualize the single-cell expression profiles [44].

### Cell type determination

To identify highly variable genes, filters were applied. Genes with normalized expression between 0.125 and 3 and expression variance in the top 50% after quantile normalization were selected. Principal component analysis (PCA) was then performed and the top 20 principal components capturing the major sources of variability were selected. These principal components were used to generate a 2D representation of the cells by t-distributed stochastic neighbor embedding (t-SNE) dimensionality reduction with default parameters. Finally, the cell types in the t-SNE plot were annotated by identifying canonical marker genes corresponding to known biological cell types.

### Statistical analysis

All experiments were performed in at least three independent biological replicates and the results are presented as mean ± standard deviation. Statistical comparisons between two experimental groups were done using two-tailed Student’s *t* tests. For comparisons of multiple groups, one-way ANOVA was performed followed by Dunnett’s post-hoc test. Kaplan–Meier survival analysis was conducted and statistical significance was determined by log-rank test. All statistical tests were carried out using either SPSS 22.0 or GraphPad Prism 7 software. A p-value less than 0.05 was considered statistically significant.

## Results

### ARNT is significantly upregulated in GBM patients

**Fig. 1:**
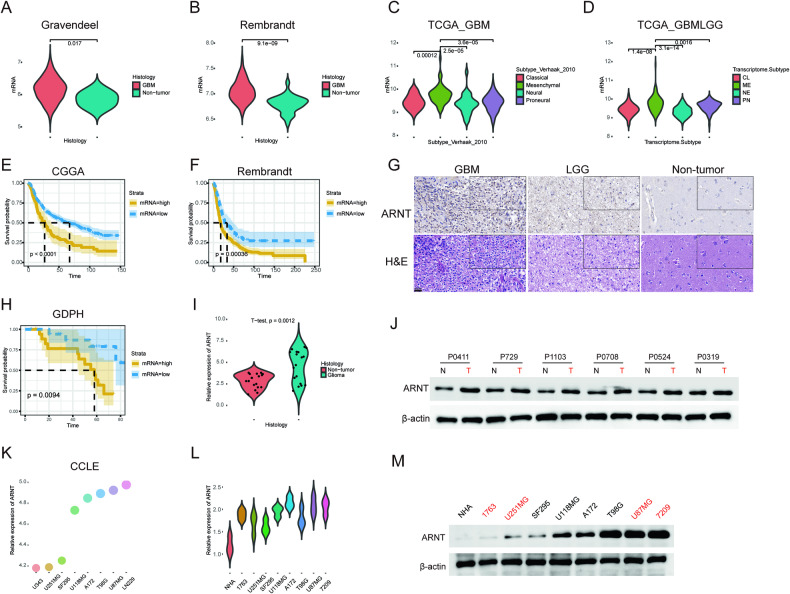
ARNT is significantly upregulated in GBM patients. **A** Analysis of ARNT gene expression in GBM versus non-tumor tissue using the Gravendeel database. Expression was compared between groups using the Student’s *t* test. **B** Analysis of ARNT gene expression in GBM versus non-tumor tissue using the Rembrandt database. Expression was compared between groups using the Student’s *t* test. **C** Analysis of ARNT gene expression in different WHO molecular GBM subtypes using the TCGA_GBM database. Expression was compared between subtypes using the Wilcoxon test. **D** Analysis of ARNT gene expression in GBM versus lower grade gliomas using the TCGA_GBMLGG database. Expression was compared between groups using the Wilcoxon test. **E** Kaplan–Meier survival analysis of GBM patients stratified by ARNT expression using the CGGA database. Groups were compared using the log-rank test. **F** Kaplan–Meier survival analysis of GBM patients stratified by ARNT expression using the Rembrandt database. Groups were compared using the log-rank test. **G** Representative immunohistochemistry images of ARNT staining in glioma samples from the GDPH Glioma database. Epilepsy brain tissue was used as a negative control. **H** Kaplan–Meier survival analysis of GBM patients stratified by ARNT mRNA level obtained from patients’ samples. **I** qRT-PCR analysis of ARNT mRNA expression in glioma tumors versus adjacent tissue using the GDPH Glioma database. Expression was compared between groups using the Student’s *t* test. **J** Western blot analysis of ARNT protein levels in patient tissue samples. β-actin was used as a loading control. **K** Analysis of ARNT gene expression in GBM cell lines from the CCLE database. **L** qRT-PCR analysis of ARNT mRNA expression in primary GBM cells, commercial GBM lines, and normal human astrocytes (NHA). **M** Western blot analysis of ARNT protein levels in cultured cell lines. β-actin was used as a loading control.

To investigate if *ARNT* is involved in the malignant progression of glioma, its mRNA expression levels were examined using several public datasets representing different pathological grades, including Gravendeel, Rembrandt, and TCGA. The results demonstrated that *ARNT* expression was significantly higher in GBM compared to non-tumor tissues (Fig. 1A, B). As is well known, the mesenchymal subtype represents a more aggressive and invasive state in glioblastomas, conferring greater chemoresistance and radioresistance [39]. Therefore, to confirm the role of *ARNT* in glioma, ARNT mRNA expression was analyzed across different molecular subtypes using TCGA datasets. As shown in Fig. 1C, D, *ARNT* was upregulated in the mesenchymal subtype of glioma, indicating that *ARNT* may serve as a regulator of chemoresistance. Furthermore, Kaplan-Meier survival analyses were performed based on *ARNT* expression, revealing that high *ARNT* expression was negatively correlated with patient outcomes (Fig. 1E, F). To gain a more profound insight into the clinical significance of *ARNT*, we conducted representative IHC staining on samples obtained from patients who underwent surgical resection at Guangdong Provincial Hospital in recent years. The results revealed a significant increase in *ARNT* expression in glioblastoma (GBM) compared to low-grade glioma or non-brain tumor tissues (Fig. 1G). Furthermore, mRNA and protein extracted from these samples were analyzed through qRT-PCR and Western blotting assays. Remarkably, patients with higher *ARNT* mRNA levels experienced a shortened lifespan, as indicated by Kaplan-Meier survival analyses (Fig. 1H). As depicted in Fig. 1I, J, *ARNT* expression was elevated in tumor samples compared to adjacent non-tumor tissues. Subsequently, we assessed *ARNT* expression levels in primary cells and commercial human GBM cell lines using CCLE public databases and experimental validation. As shown in Fig. 1K–M, 1763 and U251MG exhibited lower endogenous *ARNT* expression, while 7209 and U87MG demonstrated higher endogenous *ARNT* expression. Consequently, these four cell lines, each with varying *ARNT* expression, were selected for subsequent experiments.

### Silencing ARNT attenuates the malignancy of glioma cell lines

**Fig. 2:**
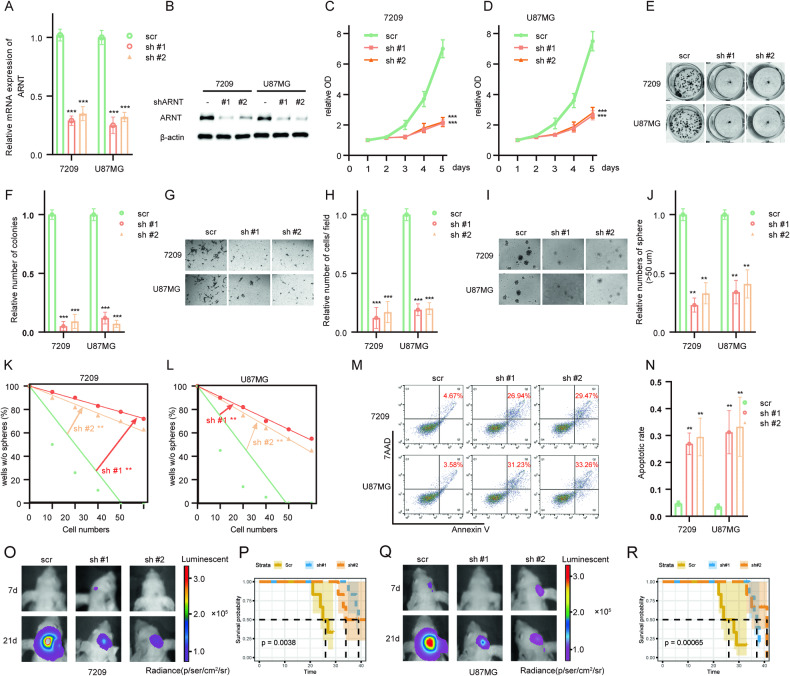
Silencing ARNT attenuates the malignancy of glioma cell lines. **A** qRT-PCR analysis of ARNT mRNA expression in 7209 and U87MG cells transfected with lentiviral shARNT #1, #2 or negative control. **B** Western blot analysis of ARNT protein expression in 7209 and U87MG cells transduced with lentiviral shARNT #1, #2 or control. **C**, **D** CCK8 proliferation assays of 7209 and U87MG cells after ARNT knockdown. **E**, **F** Colony formation assays of 7209 and U87MG cells after transduction with lentiviral shARNT #1, #2 or control. **G**, **H** Transwell invasion assays of 7209 and U87MG cells transduced with lentiviral shARNT #1, #2 or control. **I**, **J** Sphere formation assays of 7209 and U87MG cells after ARNT knockdown. **K**, **L** Limited dilution assays were performed on 7209 and U87MG cells transduced with lentiviruses expressing shARNT #1, shARNT #2, or negative control shRNA. **M**, **N** Flow cytometry analysis of apoptosis in 7209 and U87MG cells transduced with lentiviral shARNT #1, #2 or negative control. Cells were stained with Annexin V and propidium iodide. **O** Luciferase-expressing 7209 cells, with or without ARNT depletion, were intracranially injected into nude mice (*n* = 6 for each group). The luminescence intensity of tumors in representative mice at the indicated time points is depicted. **P** Kaplan–Meier analysis of 7209 tumor-bearing mice. **Q** Luciferase-expressing U87MG cells, with or without ARNT depletion, were intracranially injected into nude mice (*n* = 6 for each group). The luminescence intensity of tumors in representative mice at the indicated time points is depicted. **R** Kaplan–Meier analysis of U87MG tumor-bearing mice. Statistical significance was determined by Student’s *t* test, *p < 0.05 was considered significant.

To further understand the function of *ARNT* in GBM, two short hairpin RNAs (shRNAs) targeting ARNT (#1 and #2) were introduced into the 7209 and U87MG cell lines via lentivirus. Quantitative real-time PCR (qRT-PCR) analysis showed that *ARNT* mRNA expression was reduced by over 50% in both cell lines transfected with shARNT lentivirus compared to scramble control (Fig. 2A). Consistently, protein levels of *ARNT* were also considerably decreased by exogenous lentiviral knockdown, as shown by Western blot (Fig. 2B). Moreover, in vitro cell viability and colony formation assays were performed to investigate the effect of *ARNT* knockdown on tumor cell proliferation. As illustrated in Fig. 2C–F, *ARNT* silencing markedly reduced the in vitro growth of 7209 and U87MG cells. Furthermore, Matrigel invasion assays were utilized to evaluate the functional role of *ARNT* on the invasive capability of GBM cells. The number of invasive cells was decreased in ARNT-knockdown cells compared to scramble control cells (Fig. 2G, H). Additionally, sphere formation and limited dilution assays revealed inhibition in both *ARNT* knockdown GBM cell lines (Fig. 2I–L). The apoptotic rates of GBM cells carrying different lentiviruses were measured by flow cytometry, showing a significant increase in apoptosis when transfected with lentiviral shARNT. Besides, an in vivo xenograft mice model was employed to investigate the impact of *ARNT* on tumorigenesis. As depicted in Fig. 2O–R, the depletion of *ARNT* resulted in a reduction in tumor growth and an extension of the lifespan of tumor-bearing mice. Also, the proliferative markers were significantly downregulated upon *ARNT* depletion (Supplementary Fig. 1). Collectively, these findings underscore the critical role of *ARNT* in GBM tumorigenesis, both in vivo and in vitro, and highlight that silencing *ARNT* mitigates the malignancy of glioma cell lines.

### ARNT overexpression enhances TMZ resistance in GBM

**Fig. 3:**
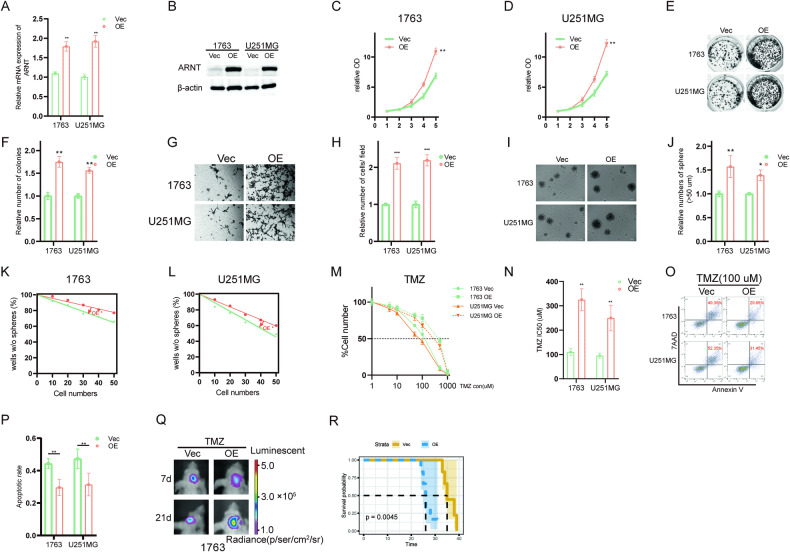
ARNT overexpression enhances TMZ resistance in GBM. **A** qRT-PCR analysis of ARNT mRNA expression in 1763 and U251MG cells transduced with lentiviral ARNT or empty vector control. **B** Western blot analysis of ARNT protein levels in 1763 and U251MG cells after transduction with lentiviral ARNT or control vector. **C**, **D** CCK8 proliferation assays of 1763 and U251MG cells after ARNT overexpression. **E**, **F** Colony formation and sphere formation assays of 1763 and U251MG cells transduced with lentiviral ARNT or control vector. **G**, **H** Transwell invasion assays of 1763 and U251MG cells after ARNT overexpression. **I**, **J** Sphere formation assays of 1763 and U251MG cells transduced with lentiviral ARNT or control vector. **K**, **L** Limited dilution assays of 1763 and U251MG cells after ARNT overexpression. **M**, **N** Dose-response curves of 1763 and U251MG cells treated with TMZ after transduction with lentiviral ARNT or control vector. Cell viability was assessed by CCK8 assay. **O**, **P** Flow cytometry analysis of apoptosis in 1763 and U251MG cells transduced with lentiviral ARNT or control vector. Cells were stained with Annexin V and propidium iodide. **Q** Luciferase-expressing 1763 cells, with or without ARNT overexpression, were intracranially injected into nude mice (*n* = 6 for each group), then treated with 30 mg/kg of TMZ. The luminescence intensity of tumors in representative mice at the indicated time points is depicted. **R** Kaplan–Meier analysis was performed on 1763 tumor-bearing mice. Statistical significance was determined by one-way ANOVA followed by Dunnett’s post-test, *p < 0.05 was considered significant.

To further investigate the function of *ARNT* in GBM cell lines, lentiviral vectors expressing *ARNT* (OE) or empty vector control (Vec) were introduced into the 1763 and U251MG cell lines. As shown in Fig. 3A, B, *ARNT* protein levels were increased over 70% in the ARNT OE cell lines compared to the control. Furthermore, in vitro cell viability and colony formation assays were performed, and the results showed that the proliferative ability of GBM cells was significantly enhanced in the *ARNT* OE groups. Matrigel invasion assays demonstrated that the invasive ability of *ARNT* OE cell lines was increased compared to vector control (Fig. 3G, H). Additionally, sphere formation and limited dilution assays were used to assess the self-renewal capacity of GBM cells. As depicted in Fig. 3I–L, the *ARNT* OE cell lines exhibited increased self-renewal ability. Moreover, these cell lines carrying different lentiviruses were treated with distinct concentrations of temozolomide (TMZ). Cell viability assays showed elevated TMZ IC50 values in the *ARNT* OE cell lines compared to vector control groups (Fig. 3M, N). The apoptotic rates of *ARNT* OE cell lines treated with TMZ were considerably decreased compared to the corresponding control groups (Fig. 3O, P). Notably, the in vivo xenograft mice model revealed that the overexpression of *ARNT* induced tumor growth and significantly desensitized the brain tumors to TMZ treatment (Fig. 3Q, R). Also, the proliferative markers were remarkably upregulated upon *ARNT* overexpression (Supplementary Fig. 2). Collectively, these results provide clear evidence that *ARNT* overexpression enhances GBM malignancy and increases chemoresistance.

### Functional roles of ARNT depicted in single-cell RNA sequencing

**Fig. 4:**
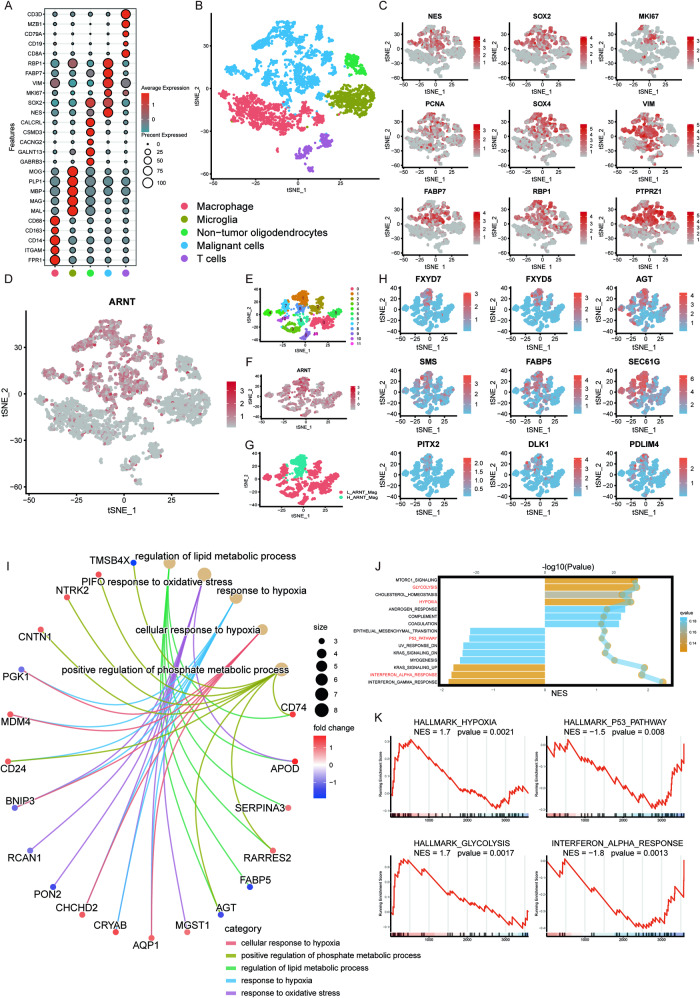
Functional roles of ARNT depicted in single-cell RNA sequencing. **A** Bubble plot showing differentially expressed genes across indicated cell clusters. **B** t-SNE plot depicting spatial distribution of various cell types in tumor and adjacent cells, colored by identity. **C** Expression of cancer stem cell markers in distinct clusters. **D** ARNT gene expression across clusters. **E** Clustering of malignant cells. **F** ARNT gene expression in relation to tumor malignancy across all malignant cells. **G** t-SNE plot dividing malignant cells into two categories based on ARNT expression. **H** Top 9 differentially expressed genes with highest expression in ARNT-high malignant cells. **I** Gene ontology analysis of differentially expressed genes in the ARNT-upregulated group. **J** Bubble plots of significantly enriched signaling pathways in malignant cells. **K** GSEA showing positive correlation between ARNT and hypoxia/glycolysis pathways and negative correlation with p53/alpha-response pathways.

Single-cell RNA sequencing (scRNA-seq) analysis was performed to elucidate the functional roles of *ARNT* in GBM at the individual cell level. Clustering analysis of scRNA-seq data revealed distinct cell subpopulations within the GBM tumor microenvironment (Fig. 4A, B). Comparison of *ARNT* expression levels across these clusters identified enrichment in a subpopulation of cancer stem-like cells (Fig. 4C, D). Malignant cells expressing higher levels of *ARNT* were re-clustered to gain deeper insights into ARNT’s functions in GBM malignancy. As shown in Fig. 4E–H, malignant cells were further classified into 2 groups. Gene ontology analysis of differentially expressed genes in ARNT-high versus ARNT-low clusters implicated *ARNT* in regulating metabolic processes, hypoxia response, and response to oxidative stress (Fig. 4I). Gene set enrichment analysis (GSEA) indicated that ARNT was involved in regulating glycolysis, hypoxia, the p53 pathway, and interferon alpha response, suggesting ARNT may serve as a marker of malignancy in GBM. Altogether, scRNA-seq analysis provides multiple lines of evidence that ARNT functions to promote glycolysis, hypoxic adaptation, stem cell propagation, and is involved in the negative regulation of the p53 pathway and interferon alpha response in GBM.

### ARNT regulates p38/MAPK signaling

**Fig. 5:**
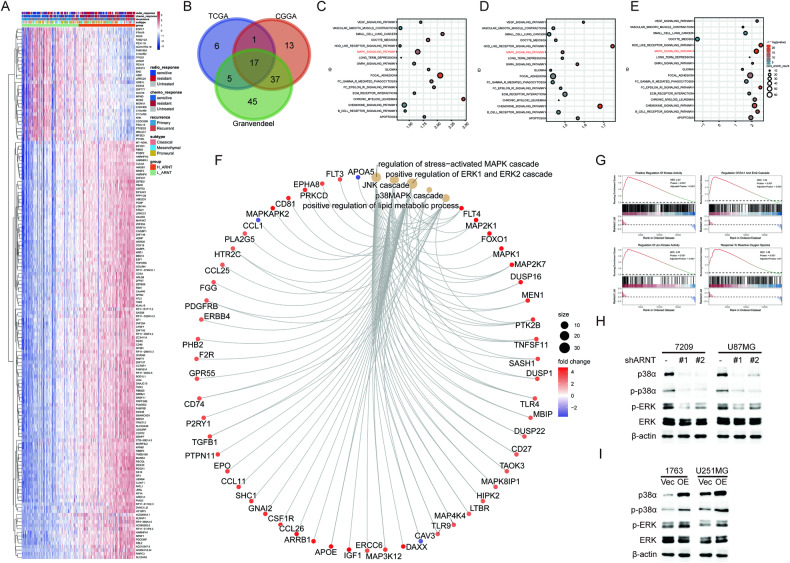
ARNT regulates p38/MAPK signaling. **A** Hierarchical clustering analysis of differentially expressed genes in ARNT-high versus ARNT-low GBMs. **B** Venn diagram showing 17 overlapping signaling pathways correlated with high ARNT expression and tumor malignancy. **C**–**E** GSEA enrichment plots using transcriptome data from CGGA, TCGA, and Gravendeel GBM cohorts. **F** Gene ontology analysis of differentially expressed genes from RNA-seq of 1763-ARNT overexpressing cells versus control. **G** GSEA analysis of 1763-ARNT RNA-seq data showing positive correlation between ARNT and p38/MAPK signaling. **H** Western blots of p38, p-p38, p-ERK, and total ERK in 7209 and U87MG cells after shARNT knockdown. **I** Western blots of p38, p-p38, p-ERK, and total ERK in 1763 and U251-ARNT overexpressing cells versus control. β-actin was used as a loading control.

To investigate the mechanisms underlying ARNT’s promotion of GBM malignancy, CGGA glioma datasets were analyzed to illustrate the correlation between *ARNT* expression and clinical significance. Samples with higher *ARNT* expression were enriched in the chemoresistance and recurrence groups, as shown in Fig. 5A. Furthermore, gene set enrichment analysis (GSEA) was performed to examine pathways associated with *ARNT* expression. The MAPK signaling pathway was significantly correlated with *ARNT* expression (Fig. 5B–E). Additionally, RNA sequencing analysis was conducted using ARNT-overexpressing 1763 cell lines and controls. Gene ontology analysis was performed to identify possible GO terms correlating with *ARNT*. Differentially expressed genes from ARNT-high and ARNT-low groups were enriched for regulation of stress-activated MAPK cascades, positive regulation of ERK1/2 cascades, p38 MAPK cascades, and positive regulation of lipid metabolic processes (Fig. 5F). Moreover, GSEA showed a positive correlation between *ARNT* and p38/MAPK-related pathways (Fig. 5G). As illustrated in Fig. 5H, I, *ARNT* knockdown considerably downregulated MAPK signaling, while *ARNT* overexpression activated the p38/MAPK pathway. Overall, these results demonstrate that *ARNT* regulates the p38/MAPK pathway to promote malignant phenotypes in GBM cells.

### Hypoxia induces TMZ resistance in a p38α/MAPK-dependent manner

**Fig. 6:**
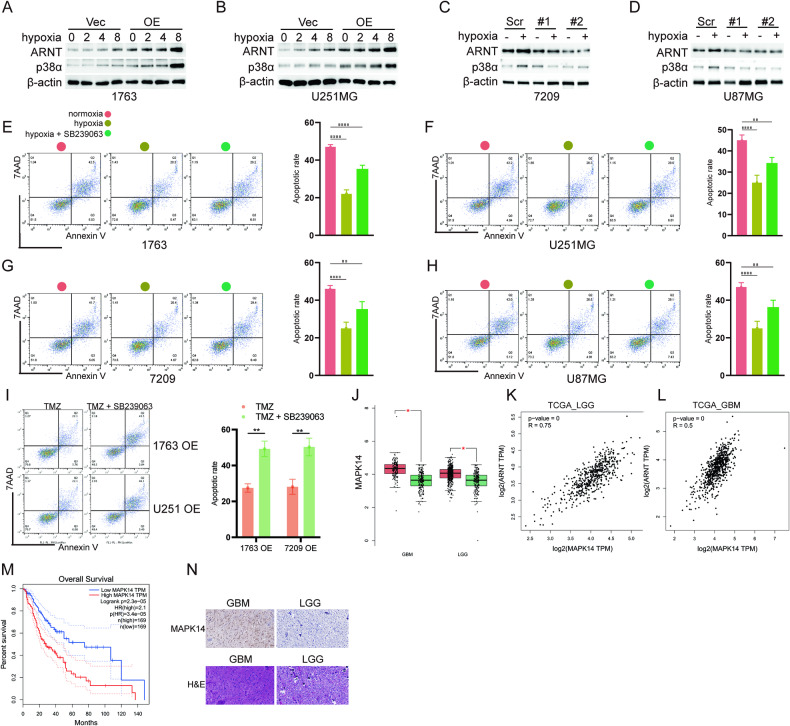
Hypoxia induces TMZ resistance in a p38α/MAPK-dependent manner. **A**, **B** Western blots showing ARNT and p38α expression in 1763 and U251-ARNT overexpression cells versus vector control under hypoxia. **C**, **D** Western blots showing ARNT and p38α expression in 7209 and U87-shARNT cells versus control under normoxia or hypoxia (8 h). **E**–**H** Flow cytometry analyzing annexin V/propidium iodide staining and apoptosis in 1763, U251, 7209, and U87 cells after TMZ treatment under normoxia, hypoxia, or hypoxia + p38 inhibitor SB239063. **I** Flow cytometry analyzing annexin V/propidium iodide staining and apoptosis in 1763 and U251 overexpression with or without p38 inhibitor SB239063. **J** MAPK14 gene expression in GBM, LGG, and normal tissue from GEPIA database (*p < 0.05, Student’s *t* test). **K**, **L** Correlation between MAPK14 and ARNT expression analyzed by GEPIA online tool (*R* = Pearson correlation coefficient). **M** Kaplan-Meier analysis of MAPK14 in TCGA-GBMLGG database from GEPIA. **N** Representative ARNT IHC images in GBM and LGG from GDPH glioma samples.

As is well known, *ARNT* is involved in regulating the hypoxia response [12]. To further understand the correlation between hypoxia and TMZ resistance in GBM cells, different cell lines carrying indicating lentiviruses were treated with CoCl_2_, a known hypoxia-mimetic agent. Both *ARNT* and *p38α* expression were markedly elevated in 1763 and U251MG glioma cell lines (*ARNT* was overexpressed as indicated) in response to CoCl_2_ (Fig. 6A, B and Supplementary Fig. 3A, B). However, upregulation of *p38α* was attenuated in *ARNT* knockdown cell lines, indicating the increase in *p38α* under hypoxic conditions (8 h with 100 µM CoCl_2_) relies on *ARNT* expression (Fig. 6C, D and Supplementary Fig. 3C, D). Moreover, apoptosis assays determined by flow cytometry showed that hypoxia reduced chemosensitivity of glioma cells to TMZ, while drug tolerance induced by hypoxia was rescued by SB239063, a p38/MAPK inhibitor (Fig. 6E–I) [40]. Furthermore, analysis of TCGA datasets revealed *p38α* (MAPK14) was upregulated in glioma samples compared to non-tumor samples (Fig. 6J). Additionally, *p38α* expression positively correlated with *ARNT* levels, while negatively correlating with patient survival (Fig. 6K–N). Altogether, these findings suggest hypoxia induces TMZ resistance via p38α/MAPK signaling in a manner dependent on *ARNT*. The aberrant elevation of *p38α* is associated with poorer patient outcomes.

### Disrupting the ARNT/p38α protein complex attenuates chemoresistance in GBM cells

**Fig. 7:**
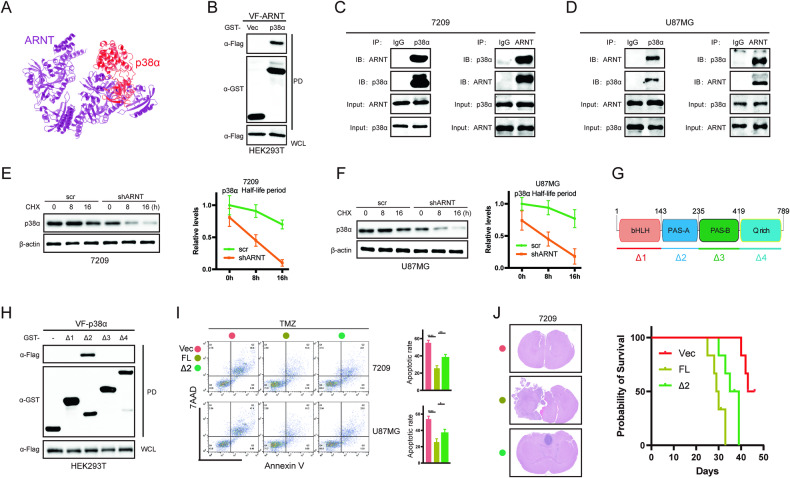
Disrupting ARNT/p38α protein complex attenuates chemoresistance in GBM cells. **A** In silico modeling of ARNT (purple) in complex with p38α (red) by Cluspro. **B** GST-pulldown assay validating ARNT-p38α interaction. **C**, **D** Co-immunoprecipitation using ARNT or p38α antibodies in 7209 and U87MG cells. Nonspecific IgG used as negative control. **E**, **F** p38α protein levels in 7209 and U87MG cells pretreated with or without shARNT after cycloheximide (CHX) treatment. β-actin was a loading control. **G** Schematic of ARNT protein structural domains. **H** GST-pulldown assay verifying interaction of ARNT domains with p38α. **I** Flow cytometry analyzing 7209 and U87MG cells after TMZ treatment and transduction with vector, full-length ARNT, or ARNT PAS-A domain. **J** Left panel H&E images of mouse brain sections after intracranial xenograft transplantation under different conditions. Right panel Kaplan–Meier survival analyses of the xenograft mouse model under the indicated conditions.

To verify the regulation of the p38/MAPK signaling pathway via *ARNT*, in silico protein-protein docking screening was performed using the ClusPro online server [45]. The protein data bank (PDB) IDs 6HWT and 4ZPH, representing p38α and ARNT respectively, were submitted to the ClusPro server. As shown in Fig. 7A, the binding model between the two proteins exhibited the lowest energy score. GST-pull down assays in HEK293T cells further confirmed p38α and ARNT form a protein complex (Fig. 7B). Additionally, co-immunoprecipitation in 7209 and U87MG glioblastoma cell lines clearly showed endogenous *ARNT* interacts with *p38α* to form a protein complex (Fig. 7C, D). CHX chase assays using 7209 and U87MG cells revealed *ARNT* knockdown decreases *p38α* protein stability (Fig. 7E, F), indicating *ARNT* regulates *p38α* levels. GST-pull down assays identified the PAS-A domain of ARNT mediates the binding to *p38α* (Fig. 7G, H). Furthermore, apoptosis assays demonstrated the *ARNT* PAS-A domain rescues chemoresistance caused by *ARNT* overexpression (Fig. 7I). An in vivo xenograft model validated the *ARNT* PAS-A domain prolongs survival of tumor-bearing mice by inhibiting glioma cell proliferation (Fig. 7J). In summary, these results demonstrate *ARNT* interacts with *p38α* to form a protein complex that activates p38/MAPK signaling. Disrupting ARNT/p38α interaction via the *ARNT* PAS-A domain attenuates chemoresistance in glioblastoma cells.

## Discussion

Glioblastoma (GBM) is one of the most lethal tumors in the human central nervous system [46, 47]. Current standard therapy consists of surgical resection followed by radiotherapy and chemotherapy with temozolomide. Despite the development of these clinical strategies over the decades, the 5-year survival rate of GBM patients is still less than 5% due to the strong proliferation and invasion ability and the acquired resistance to radiotherapy and chemotherapy [48, 49]. Previous studies identified that gene-targeted therapy for the molecular mechanism of tumorigenesis is gradually turning into a new direction for tumor therapy [50]. Therefore, developing new biomarkers and therapeutic strategies targeting GBM is in urgent need.

Accumulating evidence has demonstrated that hypoxia-inducible factors play a vital role in the progression, metastasis, treatment resistance, and prognosis of various tumors [51]. Targeting HIF activity has been verified as an effective way to reduce tumor growth [52, 53]. The Aryl Hydrocarbon Receptor Nuclear Translocator (ARNT) is a characterized transcriptional regulator mainly located in the nucleus. It forms heterodimers with several major regulatory proteins and stably regulates them. ARNT promotes organogenesis and neural development [54]. Ectopic expression of ARNT leads to attenuated physiological adaptation to hypoxia and pollutants, and promotes the initiation and progression of cancerous cells [54, 55]. Accumulating evidence reveals that ARNT is upregulated in a variety of cancer tissues and cell lines, indicating the involvement of ARNT in tumorigenesis. For example, a recent study demonstrated that HIF-1α is transported to the nucleus where it dimerizes with HIF-1β, resulting in promotion of cell migration, proliferation, invasion and tumor growth in ovarian cancer [56]. Although ARNT has been confirmed as an oncogene playing a critical role in various cancers, its functional role in glioblastoma multiforme (GBM) has not been fully elucidated. In this study, we analyzed transcriptome expression profiles from five published databases related to GBM and verified that ARNT expression was significantly upregulated in GBM patients. Moreover, we identified that elevated ARNT expression closely correlated with poor prognosis in glioma patients. Next, molecular biological assays were performed to evaluate the potential role of ARNT in GBM using lentiviral silencing and overexpression. The results showed that suppressing ARNT attenuated the malignancy of GBM cells, while ARNT overexpression promoted migratory and invasive abilities in GBM cells. Additionally, high-throughput RNA sequencing followed by gene set enrichment analysis (GSEA) and gene ontology (GO) analysis suggested that ARNT positively correlated with the MAPK signaling pathway. Subsequently, molecular biological assays proved that ARNT was a critical regulator that bound to p38α to form a protein complex, thus increasing p38α stability and inducing activation of the MAPK signaling pathway.

p38 Mitogen-Activated Protein Kinases (p38 MAPKs) includes four isoforms: p38α (MAPK14), p38β (MAPK11), p38γ (MAPK12), and p38δ (MAPK13) [57]. p38α is expressed in all cell types and tissues. In resting cells, p38α/β are mainly found in the cytoplasm, with certain molecules undergoing phosphorylation in response to stimulation [57]. Whether phosphorylated or not, p38α/β form a complex with either a dimer of Imp7/3 or Imp9/3, which transport them to the nuclear pores. While Imp3 remains outside, Imp7 or Imp9 accompany p38α/β into the nucleus. Once inside, p38α/β dissociate from the importins and proceed to phosphorylate their substrates. They are then exported back to the cytoplasm.

Phosphorylated p38 MAPK can activate a wide range of stimuli such as transcription factors, protein kinases, cytoplasmic substrates, and nuclear substrates [19]. The downstream events of phosphorylated p38 MAPK have cell-specific consequences including regulation of RNA splicing, cytokine production, inflammatory response, apoptosis, cell-cycle arrest, and cell differentiation [19]. Activated p38α has also been shown to support cell survival via anti-apoptotic interleukin-6 (IL-6) inflammatory signals and enable DNA repair after chemotherapy, resulting in drug resistance in cancer cells [20]. Moreover, upregulation of p38 MAPK promotes cell invasion by inducing epithelial to mesenchymal transdifferentiation (EMT) [58]. Downregulation of p38 MAPK leads to suppressed expression and activity of matrix metalloproteinases MMP-2 and MMP-9, supporting the role of p38 in facilitating cancer cell invasion [59]. Activated p38 signaling also increases cell migration via vascular endothelial growth factor (VEGF) expression, promoting actin rearrangement [60]. The studies noted above demonstrate the importance of the role of p38 MAPK signaling as an oncogene in many tumor types. Upon examining the significantly enriched GO terms as previously presented, we found that the p38-MAPK signaling pathway is particularly relevant to cancer biology, as supported by extensive literature [30, 61, 62]. Moreover, p38-MAPK is known to act as an upstream regulator of the VEGF signaling pathway [63], which is also prominently enriched in our analysis. Therefore, investigating the mechanism by which p38-MAPK modulates resistance to TMZ chemotherapy in glioma is of significant interest. To explore the specific sites where ARNT interacts with p38α to form protein complexes, we conducted experimental research on the functional domains of ARNT.

The HIF-1β subunit is the protein product of the ARNT gene, which was previously shown to encode 789 amino acid isoforms of the aryl hydrocarbon receptor (AHR) nuclear translocator protein [64]. ARNT can, therefore, dimerize with HIF-1α in cells subjected to hypoxia to form HIF-1. It can also dimerize with AHR in cells exposed to aryl hydrocarbons such as dioxin to form the AHR complex. The HIF-1β (ARNT) protein contains the following structural motifs: The bHLH, or basic helix-loop-helix domain, is the hallmark of an extensive superfamily of transcription factors. The HLH domains mediate protein dimerization that is necessary for DNA binding mediated by the basic domains. Whereas the HLH domain is sufficient for dimerization of most bHLH proteins, HIF-1β (ARNT) contains a second required dimerization domain, PAS, which was originally identified by the presence of related sequences in the PER (which does not contain a bHLH domain), ARNT, and SIM proteins [64]. All PAS domains contain two internal homology units, the A and B repeats, each of which contains an invariant HXXD motif (H, histidine; X, any amino acid; D, aspartate) [65]. For HIF-1β (ARNT), the HLH and PAS domains together create a functional interface for subunit protein-protein dimerization. The Q rich, carboxyl half of the HIF-1β (ARNT) proteins contains one or more potent transactivation domains which are presumed to interact directly or indirectly with components of the transcription initiation complex and thus affect the rate of transcription of genes to which they have bound. In this study, we designated each functional domain of ARNT with bHLH as ∆1, PAS-A as ∆2, PAS-B as ∆3, and Q rich as ∆4. Moreover, our experimental results showed that the truncation of ARNT, PAS-A (△2), can form a protein complex with p38α and competitively inhibit the protein interaction between the full-length ARNT protein and p38α.

Although previous studies have identified that the MAPK signaling pathway is involved in the progression of GBM, the correlation between MAPK and ARNT has not been assessed. Our data indicated that ARNT knockdown reduced the protein levels of p38α, while the MAPK signaling pathway is mediated by p38α. Therefore, we hypothesize that ARNT activates the MAPK pathway by interacting with p38α. To test this, GST pull down and CO-IP assays were performed. As expected, western blot assays exhibited the presence of ARNT and p38 in the affinity-purified protein complex, indicating an interaction between ARNT and p38. Moreover, we observed that the protein levels and protein stability of p38α were reduced in ARNT knockdown GBM cells compared to control GBM cells.

Taken together, our data demonstrate that ARNT induces activation of the MAPK signaling pathway by binding to p38α to form a protein complex, increasing its stability. The truncation of ARNT, PAS-A(△2), can competitively inhibit the protein interaction between full-length ARNT protein and p38α. These findings reveal a new mechanism through which ARNT leads to malignant transformation of GBM, thereby promoting the design of more effective ARNT-targeted therapies.

Although the potential role of ARNT in GBM and related pathways have been well discussed here, further research on the molecular mechanisms is still required to evaluate the clinical significance of ARNT in GBM. For instance, the upstream regulators of ARNT in GBM remain largely unknown. Although the interaction between ARNT and p38α has been verified, further molecular experiments are needed to determine whether their binding sites could affect the downstream of MAPK signaling. In addition, due to the heterogeneity of GBM, inhibition of a single candidate biomarker might have unpredictable effects. Therefore, comprehensive evaluation of GBM patients is essential before clinical management.

## Conclusion

In summary, this study provides compelling evidence that ARNT is upregulated and clinically relevant in GBM. Functional experiments demonstrate ARNT promotes proliferative, invasive, and stem cell-like capabilities in GBM cells. ARNT activates p38/MAPK signaling by interacting with p38α to induce chemoresistance. Targeting the ARNT/p38α complex represents a potential therapeutic strategy to overcome chemoresistance in GBM patients. Further research should explore combining ARNT/p38α inhibitors with conventional chemotherapy to improve outcomes for this devastating disease. This study advances our understanding of GBM pathogenesis and identifies ARNT as a critical oncogenic driver and novel therapeutic target in GBM.

### Supplementary information

WB uncropped

Supplementary Figures

## Data Availability

The authors declare that all data and material in the article is available upon request.
