# Supplementary figures and images for: Targeting ARNT attenuates chemoresistance through destabilizing p38α-MAPK signaling in glioblastoma
Source: Cell Death Dis. 2024 May 28;15(5):366. doi: 10.1038/s41419-024-06735-1 (PMC11133443; doi:10.1038/s41419-024-06735-1)

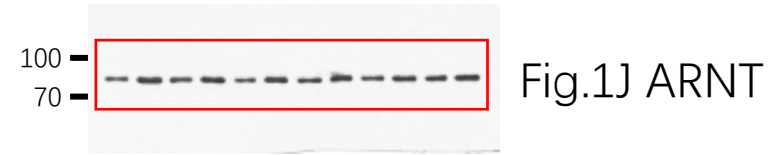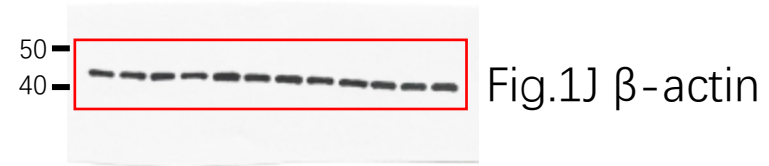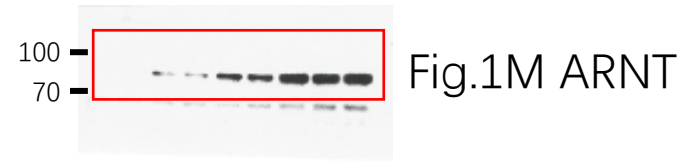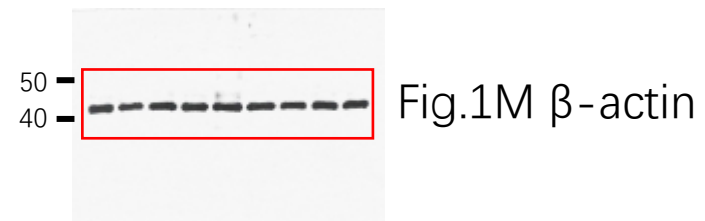

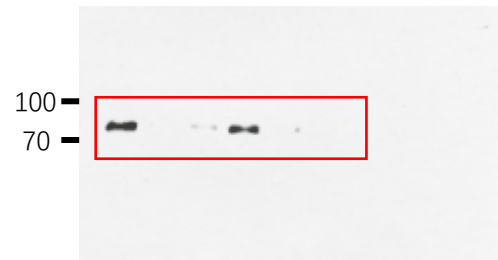

Fig.2B ARNT

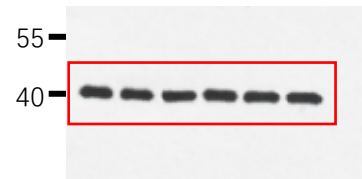

Fig.2B  $\beta$ -actin

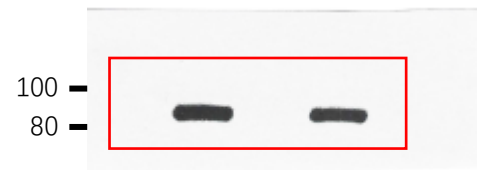

Fig.3B ARNT

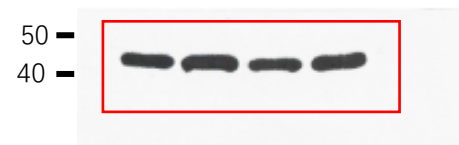

Fig.3B  $\beta$ -actin

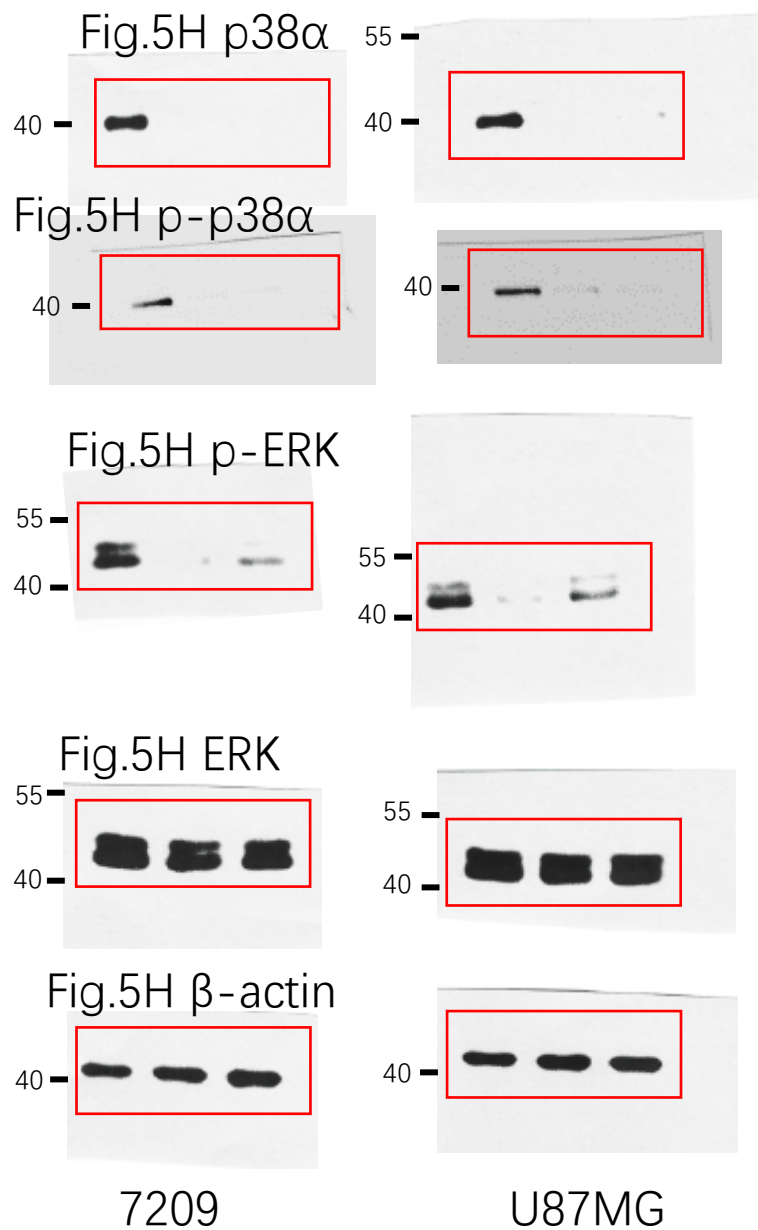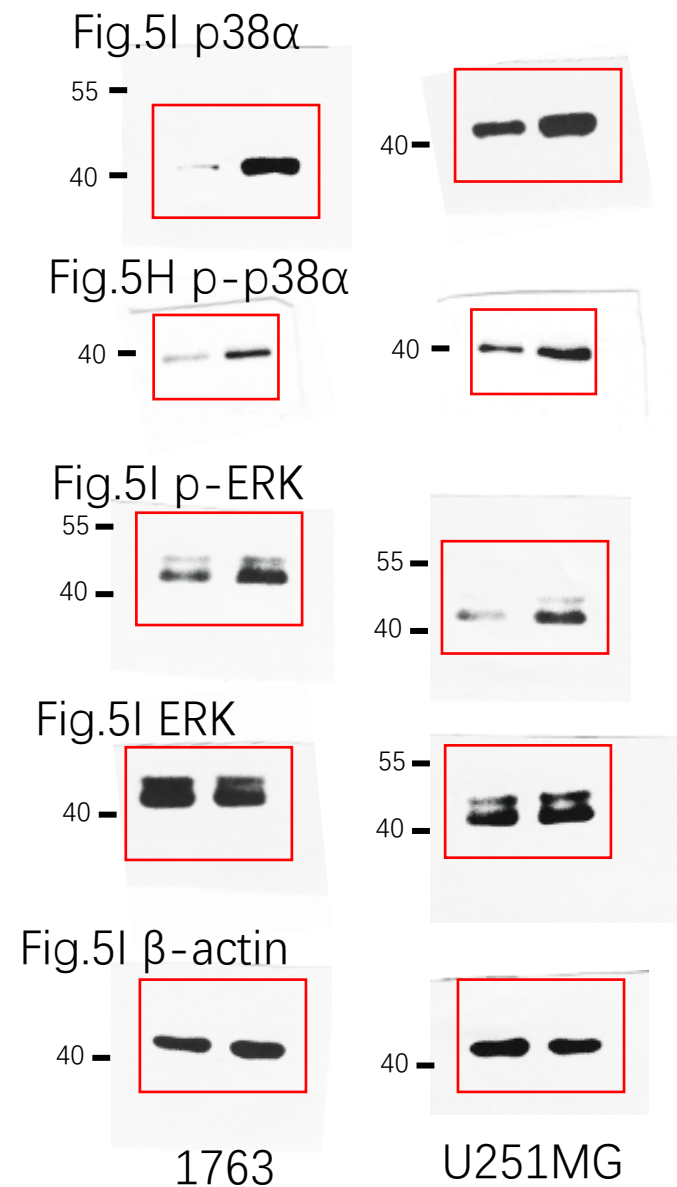

Fig.6A

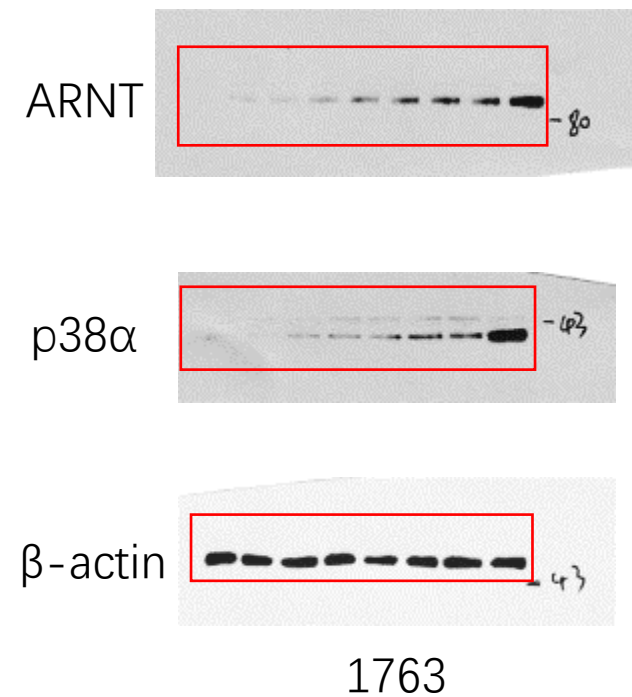

Fig.6B

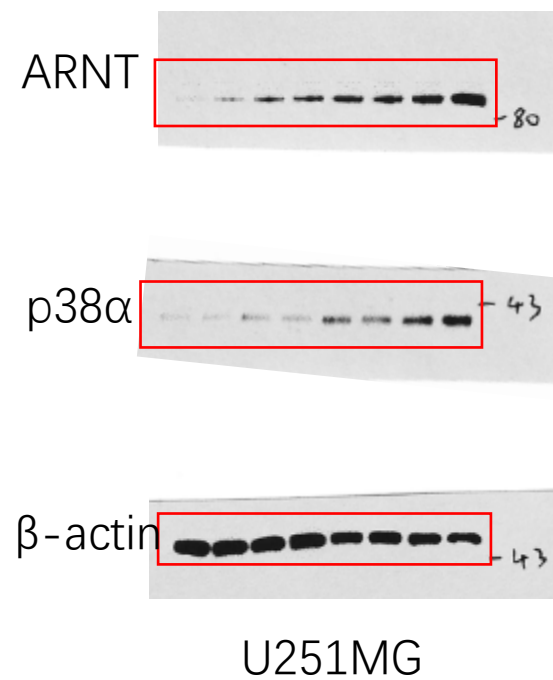

Fig.6C

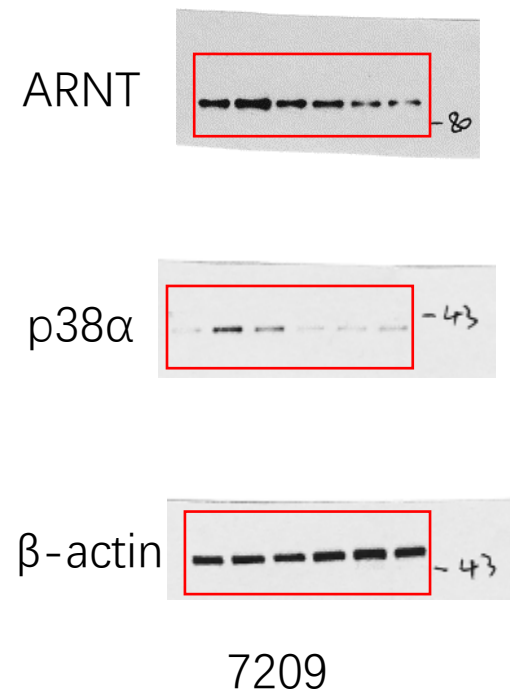

Fig.6D

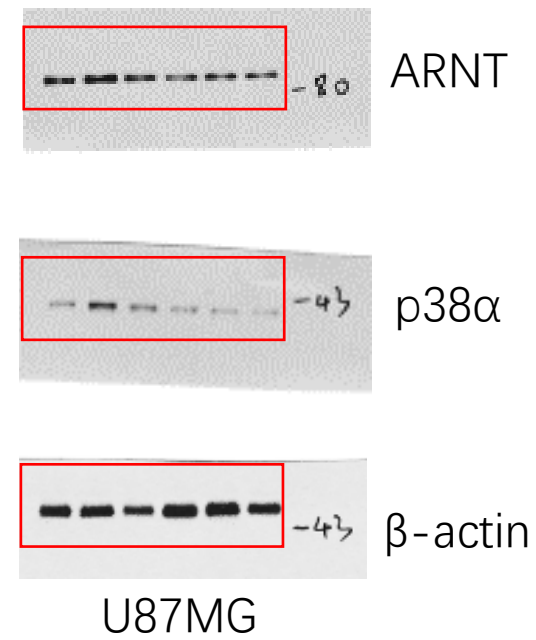

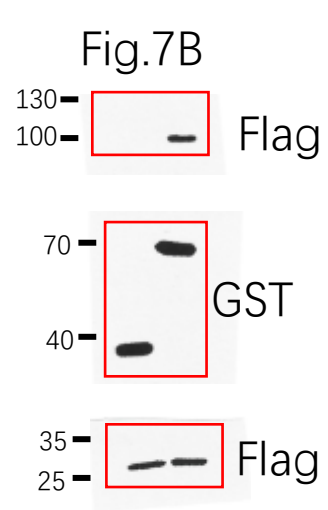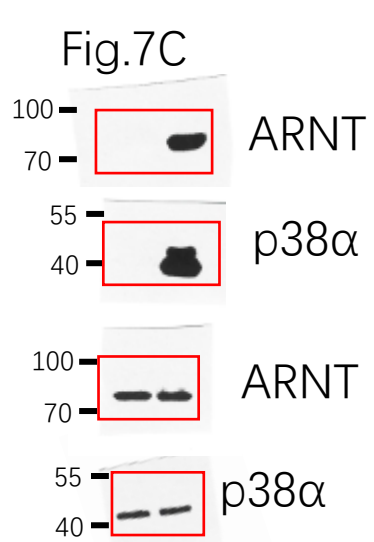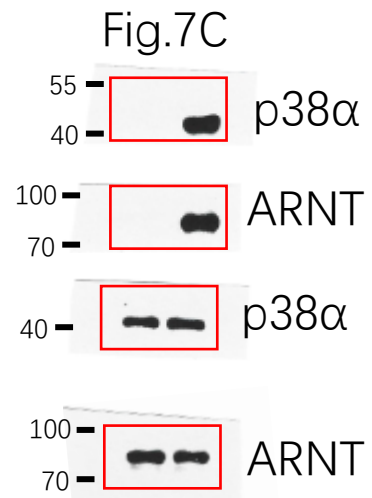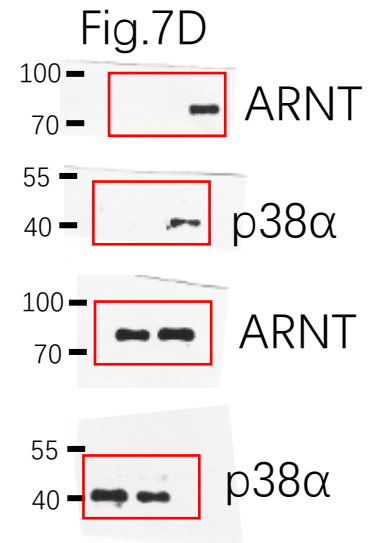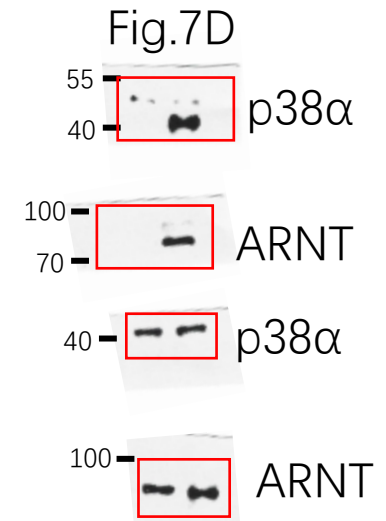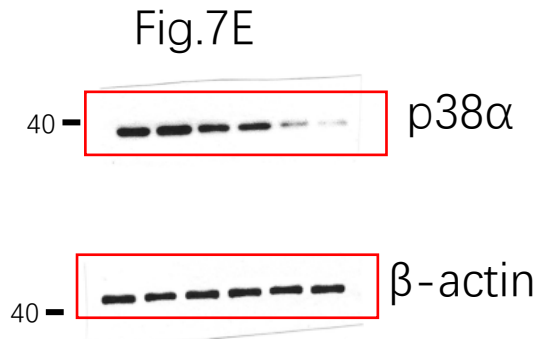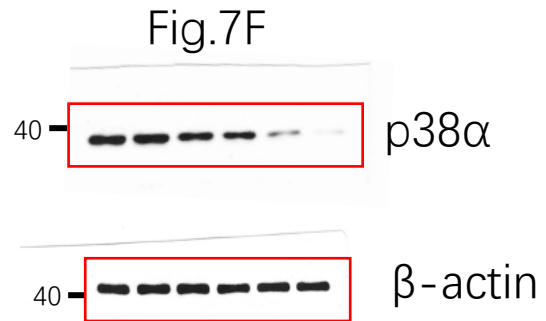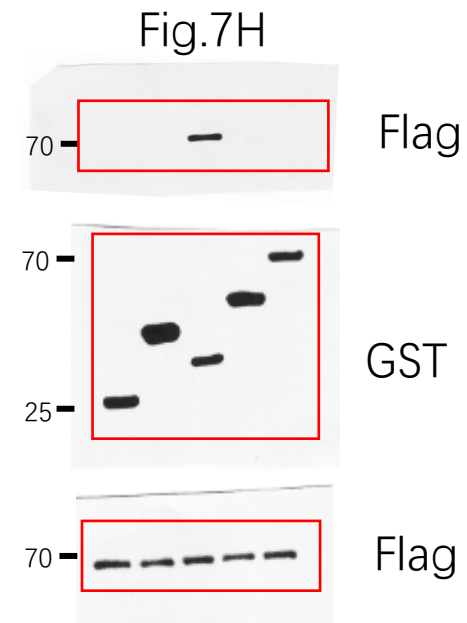

Supplement: Supplementary file 1 — WB uncropped [file 41419_2024_6735_MOESM1_ESM.pdf]
