# Supplementary material for: Targeting ARNT attenuates chemoresistance through destabilizing p38α-MAPK signaling in glioblastoma
Source: Cell Death Dis. 2024 May 28;15(5):366. doi: 10.1038/s41419-024-06735-1 (PMC11133443; doi:10.1038/s41419-024-06735-1)
Supplement: Supplementary file 2 — Supplementary Figures [file 41419_2024_6735_MOESM2_ESM.pdf]

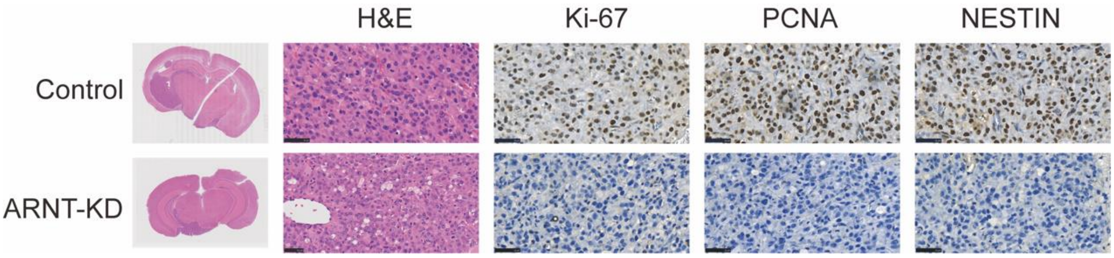

Supplementary Fig. 1. The IHC staining of Ki-67, PCNA and NESTIN were performed using *in vivo* xenograft tissues. As is shown above, the expression of proliferative markers were significantly downregulated by ARNT-knockdown.

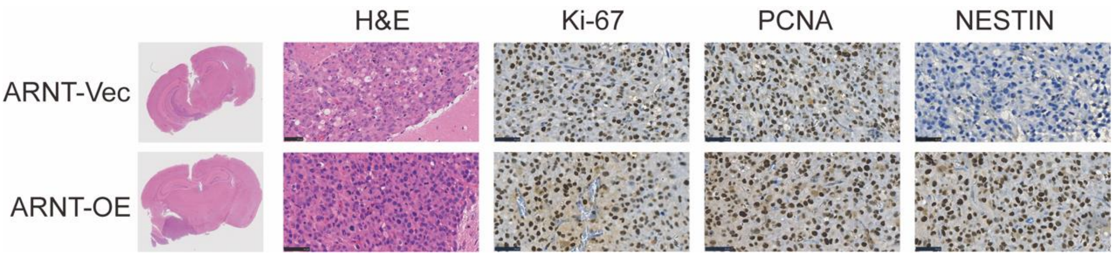

Supplementary Fig. 2. The IHC staining of Ki-67, PCNA and NESTIN were performed using *in vivo* xenograft tissues. As is shown above, the expression of proliferative markers were significantly downregulated by ARNT-overexpression.

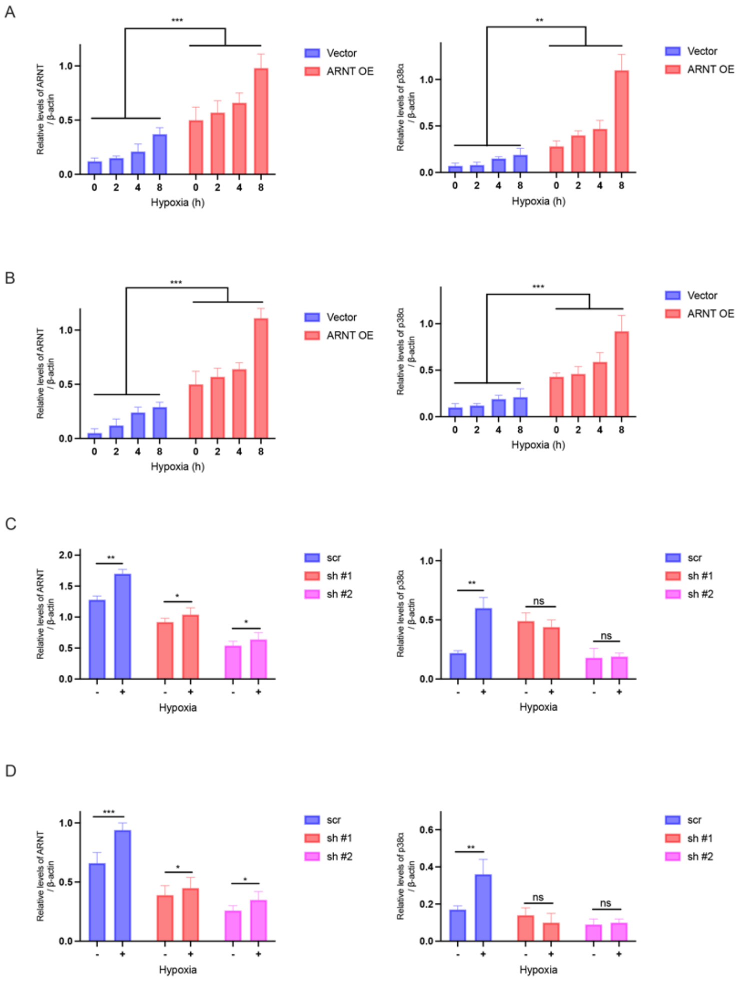

Supplementary Fig. 3. The densitometry analyses of western blot in Figure 6.A-D.
